# Dissimilatory Nitrate Reduction to Ammonium and Responsible Microbes in Japanese Rice Paddy Soil

**DOI:** 10.1264/jsme2.ME20069

**Published:** 2020-10-06

**Authors:** Yosuke Nojiri, Yuka Kaneko, Yoichi Azegami, Yutaka Shiratori, Nobuhito Ohte, Keishi Senoo, Shigeto Otsuka, Kazuo Isobe

**Affiliations:** 1 Graduate School of Agricultural and Life Sciences, The University of Tokyo, Tokyo, Japan; 2 Niigata Agricultural Research Institute, Niigata, Japan; 3 Graduate School of Informatics, Kyoto University, Kyoto, Japan; 4 Collaborative Research Institute for Innovative Microbiology, The University of Tokyo, Tokyo, Japan

**Keywords:** DNRA, denitrification, *nrfA*, *nirK*, *nirS*

## Abstract

Nitrification–denitrification processes in the nitrogen cycle have been extensively examined in rice paddy soils. Nitrate is generally depleted in the reduced soil layer below the thin oxidized layer at the surface, and this may be attributed to high denitrification activity. In the present study, we investigated dissimilatory nitrate reduction to ammonium (DNRA), which competes with denitrification for nitrate, in order to challenge the conventional view of nitrogen cycling in paddy soils. We performed paddy soil microcosm experiments using ^15^N tracer analyses to assess DNRA and denitrification rates and conducted clone library analyses of transcripts of nitrite reductase genes (*nrfA*, *nirS*, and *nirK*) in order to identify the microbial populations carrying out these processes. The results obtained showed that DNRA occurred to a similar extent to denitrification and appeared to be enhanced by a nitrate limitation relative to organic carbon. We also demonstrated that different microbial taxa were responsible for these distinct processes. Based on these results and previous field observations, nitrate produced by nitrification within the surface oxidized layer may be reduced not only to gaseous N_2_ via denitrification, but also to NH_4_^+^ via DNRA, within the reduced layer. The present results also indicate that DNRA reduces N loss through denitrification and nitrate leaching and provides ammonium to rice roots in rice paddy fields.

Rice is one of the most important agronomic products in the world. The supply of nitrogen (N) to rice roots generally limits production. Various biochemical processes involved in N cycling occur in paddy soils. As initially discovered in the 1930s, nitrification (NH_4_^+^→NO_2_^–^→NO_3_^–^) occurs within a thin oxidized soil surface layer, while denitrification (NO_3_^–^→NO_2_^–^→NO→N_2_O→N_2_) occurs within a reduced soil layer below the oxidized layer (Ishii *et al.*, 2011). Nitrification–denitrification processes form the core of the N cycle, and, thus, have been extensively examined (see reviews by Ishii *et al.*, 2011). The diverse microbes responsible for these processes were identified in the 2000s and 2010s (Ishii *et al.*, 2009; Yoshida *et al.*, 2009; Ishii *et al.*, 2010; Yoshida *et al.*, 2010; Tago *et al.*, 2011; Yoshida *et al.*, 2012; Nishizawa *et al.*, 2013; Wei *et al.*, 2015; Masuda *et al.*, 2017; 2019; Fujimura *et al.*, 2020). Since rice preferably utilizes NH_4_^+^ over NO_3_^–^ (Sasakawa and Yamamoto, 1978), the sequential processes of nitrification–denitrification that eventually lead to N loss from soil appear to reduce the efficiency of ammonium-based fertilization.

Dissimilatory nitrate reduction to ammonium (DNRA, NO_3_^–^→NO_2_^–^→NH_4_^+^) challenges our current knowledge of the N cycle in paddy soils. DNRA competes with denitrification for NO_3_^–^ and has a similar energy yield. Theoretically, DNRA yields more free energy per molecule of NO_3_^–^ reduced than complete denitrification, while denitrification yields more free energy for every electron or carbon oxidized, as shown by the equations below (Strohm *et al.*, 2007; van den Berg *et al.*, 2015).

NO_3_^–^+5/4 CH_2_O+H^+^→1/2 N_2_+5/4 CO_2_+7/4 H_2_O

(ΔG°'=–445 kJ per mol carbon, –556 kJ per mol NO_3_^–^)

NO_2_^–^+2 CH_2_O+2 H^+^→NH_4_^+^+2 CO_2_+H_2_O

(ΔG°'=–311 kJ per mol carbon, –623 kJ per mol NO_3_^–^)

Therefore, DNRA may be more favorable than denitrification in NO_3_^–^-depleted environments. Laboratory experiments with bacterial isolation or enrichment cultures possessing the capacity for DNRA and denitrification showed that conditions with a NO_3_^–^ limitation relative to organic carbon favored DNRA over denitrification (Strohm *et al.*, 2007; Stremińska *et al.*, 2012; van den Berg *et al.*, 2015).

Submerged anaerobic paddy soils are generally NO_3_^–^-depleted environments (Nishimura *et al.*, 2004; Itoh *et al.*, 2013), which is in contrast to the continuous presence of dissolved organic carbon (C) in these soils (Xu *et al.*, 2013; Said-Pullicino *et al.*, 2016). This may be due to the strong denitrification activity of these soils (Ishii *et al.*, 2011); however, DNRA may be an alternative process that depletes NO_3_^–^ in paddy soils. Denitrification functions as an N loss process. In contrast, DNRA functions as a N retention process by preventing N loss via denitrification and reducing water-leachable NO_3_^–^ to NH_4_^+^, which is more likely to be absorbed by negatively charged soil particles (Rütting *et al.*, 2011). DNRA also supplies NH_4_^+^ to rice roots in paddy soils.

DNRA has been detected in various soil environments, including forests (Zhang *et al.*, 2018), grasslands (Rütting and Müller, 2008), upland croplands (Putz *et al.*, 2018), and deserts (Yang *et al.*, 2017). However, research on DNRA in rice paddy fields remains limited, in terms of the number of studies conducted and the range of geographic locations examined (USA, China, Australia, and Malaysia) (Buresh and Patrick, 1978; Shan *et al.*, 2016; Pandey *et al.*, 2018; 2019). Furthermore, microbial populations actively carrying out DNRA in paddy fields have not been characterized in detail. In the present study, we initially investigated whether DNRA occurs in Japanese rice paddy soil and then examined the effects of a NO_3_^–^ limitation relative to organic carbon on the partitioning of NO_3_^–^ reduction between DNRA and denitrification. We then identified the microbial populations carrying out DNRA and denitrification to establish whether these processes were driven by similar or different microbial communities.

## Materials and Methods

We collected two soil samples for soil microcosm experiments. Both samples were collected from a paddy field at the Niigata Agricultural Research Institute (37°43'N, 138°87'E) in Niigata Prefecture, the richest rice-producing area in Japan. Rice (*Oryza sativa* L., cv. Koshihikari) has been cultivated in the field since 2003. The soil is classified as grey lowland soil. We sampled soil at a depth of 0–20‍ ‍cm, removed the oxidized layer (1‍ ‍cm from the top), then sieved (<4‍ ‍mm) and mixed the soil. The biogeochemical conditions of the field were previously described by Itoh *et al.* (2013). The sample collected on June 11, 2014 was reserved for the first experiment, while that collected on June 12, 2015 was used in the second and third experiments (see below). The concentrations of dissolved organic carbon and NH_4_^+^ in the sample collected on June 12, 2015 were 25.9±0.3‍ ‍μg C (g soil)^–1^ (*n*=5), as measured by a TOC meter (TOC-VCPH, Shimadzu), and 1.4±0.1‍ ‍μg N (g soil)^–1^ (*n*=5), as measured by the indophenol spectrophotometric method with a 2 M KCl extract (Isobe *et al.*, 2011b), whereas NO_3_^–^ was not detected. These data were not obtained for the sample collected on June 11, 2014.

Our first objective was to investigate the presence of DNRA in Japanese rice paddy soil. Ten grams of soil and 9.5‍ ‍mL of water were dispensed into a 50-mL glass vial. The vial was crimp-sealed using a butyl stopper and the headspace was replaced by pure helium three times. The vial was then incubated at 26°C for 48 h, during which soil NO_3_^–^ was completely depleted. We then added ^14^NH_4_^15^NO_3_ solution enriched with 99% ^15^N (SI Science) (1.6‍ ‍μmol ^15^N [g soil]^–1^) and glucose (0 [control, org-C/NO_3_^–^=0] or 11.2‍ ‍μmol C [g soil]^–1^ [org-C/NO_3_^–^=7]) using a syringe with a fine needle after the headspace was replaced by pure helium gas. The addition of ^14^NH_4_^+^ prevents underestimations of DNRA because it inhibits the microbial assimilation of ^15^NO_3_^–^ and DNRA-generating ^15^NH_4_^+^. We incubated the vial for 10‍ ‍min and 4, 8, 12, and 24 h and then measured ^15^NO_3_^–^, ^15^NO_2_^–^, ^15^NH_4_^+^, ^15^N_2_O, and ^15^N_2_ (*n*=2 for each incubation time). We initially measured ^15^N_2_O and ^15^N_2_ in the headspace with gas chromatography mass spectrometry (GCMS) according to the method described by Isobe *et al.* (2011a). Dissolved ^15^N_2_O and ^15^N_2_ were calculated based on the Bunsen coefficient (Tiedje, 1994). We then added 32‍ ‍mL of 3 M KCl to the vial, shook at 200 rpm for 1 h, and filtered the extract using a glass filter (GF/F). We measured ^15^NO_3_^–^, ^15^NO_2_^–^, and ^15^NH_4_^+^ in the extract using the method described by Isobe *et al.* (2011b). At the same time, to confirm whether NH_4_^+^ oxidation to NO_2_^–^ or NO_3_^–^occurred, we added ^15^NH_4_^14^NO_3_ solution instead of ^14^NH_4_^15^NO_3_ and then measured ^15^NO_3_^–^, ^15^NO_2_^–^, and ^15^NH_4_^+^ in the same manner. We also prepared soil microcosms for the gene transcription survey. RNA was extracted from a 2-g soil sample and purified, and cDNA was synthesized with RNA solution as previously described (Wei *et al.*, 2015). We used soil samples before the addition of ^14^NH_4_^15^NO_3_ and incubated the vials for 8 and 12 h. The gene transcripts of pentaheme nitrite reductase (NrfA), which catalyzes the last step of DNRA (NO_2_^–^→NH_4_^+^), were amplified using a polymerase chain reaction (PCR) with the primers nrfAF2aw and nrfAR1 (Welsh *et al.*, 2014). These primers and PCR conditions are listed in Table S1. The composition of the reaction mixture and the details of the procedure are described in Isobe *et al.* (2012).

Our second objective was to examine the effects of the NO_3_^–^ limitation on DNRA. We followed the same procedure as the first experiment, except for the org-C/NO_3_^–^ of ^14^NH_4_^15^NO_3_, glucose solutions, and incubation times. Org-C/NO_3_^–^ (mol/mol) ranged from 0, 1, 3, 5, and 7 to 9 by changing the concentration of added glucose (from 0, 1.6, 4.8, 8.0, and 11.2, to 14.4‍ ‍μmol ^13^C [g soil]^–1^), while maintaining that of ^14^NH_4_^15^NO_3_ (1.6‍ ‍μmol ^15^N [g soil]^–1^). We then incubated the soil microcosms for 12 h and measured ^15^NO_3_^–^, ^15^NO_2_^–^, ^15^NH_4_^+^, ^15^N_2_O, and ^15^N_2_ (*n*=2 for each org-C/NO_3_^–^) as in the first experiment. We also investigated whether NH_4_^+^ oxidation to NO_2_^–^ or NO_3_^–^ occurred by adding the ^15^NH_4_^14^NO_3_ solution and measuring changes in ^15^NO_3_^–^ and ^15^NO_2_^–^. The DNRA rate was calculated from ^15^NH_4_^+^ production during 10‍ ‍min and 12 h, whereas the denitrification rate was calculated from the production of ^15^N of N_2_ and N_2_O during 10‍ ‍min and 12 h.

Our final objective was to identify the microbial populations involved in DNRA and denitrification. We constructed clone libraries of the gene transcripts for DNRA and denitrification. RNA was extracted from the soils of org-C/NO_3_^–^=3 (see below) used in the second experiment. The *nrfA* gene transcripts were amplified by PCR using the primers nrfAF2aw and nrfAR1. The gene transcripts of copper-containing nitrite reductase (NirK) and cytochrome cd1-containing nitrite reductase (NirS), which catalyze the step of denitrification (NO_2_^–^–>NO), were also amplified using the following primers: nirKC1F and nirKC1R (*nirK* Cluster I), nirKC2F and nirKC2R (*nirK* Cluster II), and nirSC1F and nirSC1R (*nirS* Cluster I). Denitrifiers may be phylogenetically grouped based on their *nirK* and *nirS* sequences into several clusters. We previously demonstrated that denitrifiers with *nirK* Cluster I and Cluster II and *nirS* Cluster I were dominant in the soils tested (Wei *et al.*, 2015; Fujimura *et al.* 2020), and, thus, targeted these genes. The primers and PCR conditions used are described in Table S1. The composition of the reaction mixture and the details of the procedure were previously described by Wei *et al.* (2015). Cloning of the PCR amplicons and sequencing were performed as described previously (Isobe *et al.*, 2012). After removing the primer-annealing regions, nucleotide sequences were translated into amino acid sequences using EMBOSS (Rice *et al.*, 2000). We developed a reference database of *nrfA*, *nirK*, or *nirS* amino acid sequences by curating the *nrfA*, *nirK*, and *nirS* databases downloaded on February 20, 2020 from the FunGene database (Fish *et al.*, 2013). We used the BLASTP function of the BLAST+ tool (Camacho *et al.*, 2009) for the homology search using the following threshold values: E value <0.001 and percent identity >60% for the taxonomic assignment. We then constructed the phylogenetic trees of clone sequences with the reference sequences. These sequences were aligned using Clustal W version 2.0, and maximum likelihood trees were constructed using a bootstrap analysis (100 replicates) performed in MEGA X (Kumar *et al.*, 2018). We also investigated the presence of the *nrfA*, *nirK*, and *nirS* genes within the complete genome of the assigned genus using the BLASTP search based on the genome sequences deposited in the Kyoto Encyclopedia of Genes and Genomes (KEGG) GENOME database (Kanehisa *et al.*, 2017).

### Nucleotide sequence accession numbers

The nucleotide sequence data reported are available in the DDBJ Sequenced Read Archive under the accession numbers LC532390–LC532446 for *nrfA*, LC532447–LC532534 for *nirK*, and LC532535–LC532580 for *nirS*.

## Results and Discussion

### DNRA activity in paddy soil

**Fig. 1.**
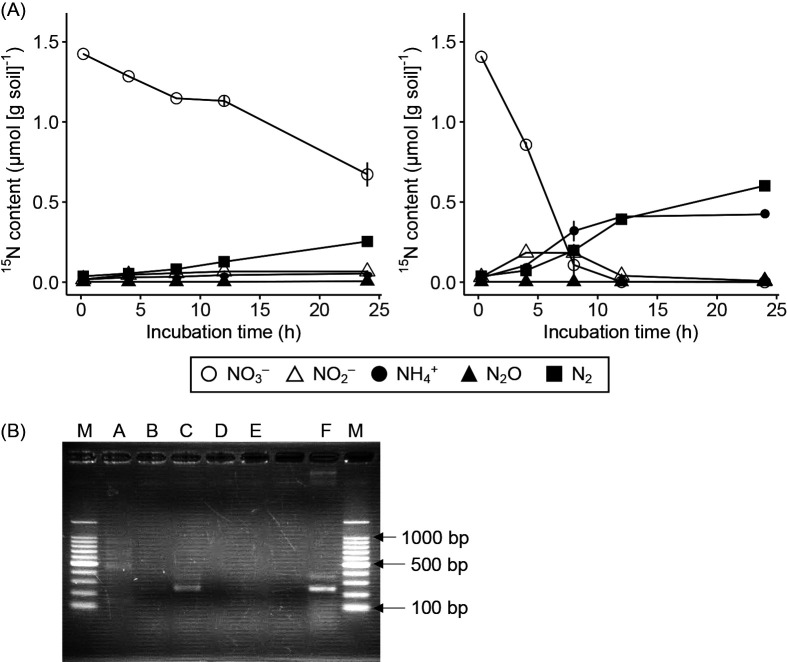
(A) Changes in ^15^N contents of NO_3_^–^, NO_2_^–^, NH_4_^+^, N_2_O, and N_2_ in the soil microcosm during a 0–24-h incubation with the addition of NH_4_^15^NO_3_ alone (left); and NH_4_^15^NO_3_ and glucose (right). The symbol represents the average, with the bar representing the range (*n*=2). (B) Detection of the *nrfA* transcript from soil by PCR. Purified RNA extracted from soil before the addition of ^15^NO_3_^–^ (Lane A), 8 h after the addition of ^15^NO_3_^–^ alone (Lane B) and ^15^NO_3_^–^ and glucose (Lane C); and 12 h after the addition of ^15^NO_3_^–^ alone (Lane D) and ^15^NO_3_^–^ and glucose (Lane E). The purified genome extracted from *Escherichia coli* was used as a positive control (Lane F). Both sides show DNA size markers (Lanes M). *E. coli* generates an approximately 270-bp amplicon of *nrfA.*

The results obtained from our first experiment confirmed that DNRA occurred in rice paddy soils at a similar rate to denitrification (Fig. 1A). NO_3_^–^ was eventually reduced to NH_4_^+^ and N_2_ in soils amended with glucose. The reduction to NO_2_^–^ was also observed after 4–12 h, whereas the concentration of N_2_O remained low around the detection limit. NO_3_^–^ reduction proceeded even without the addition of glucose and mostly led to N_2_ production. The extent of NH_4_^+^ production via DNRA with glucose during 12 h was similar to that of N_2_ production via denitrification, whereas NH_4_^+^ production via DNRA was minimal without glucose. We did not detect the oxidation of NH_4_^+^ to NO_2_^–^ or NO_3_^–^ in soil microcosms amended with ^15^NH_4_NO_3_ and glucose or with ^15^NH_4_NO_3_ alone. *nrfA* was strongly expressed in soils amended with glucose after 8 h when ^15^NH_4_^+^ was produced at the highest rate (Fig. 1B). Conversely, *nrfA* expression was less evident before NH_4_^+^ production started or when it ended (after 12 h). *nrfA* expression was also less evident in soils without glucose. These results indicate that DNRA occurred in rice paddy soils and was driven by *nrfA*-possessing microbial populations.

### Effects of the nitrate limitation relative to organic carbon on DNRA and denitrification

**Fig. 2.**
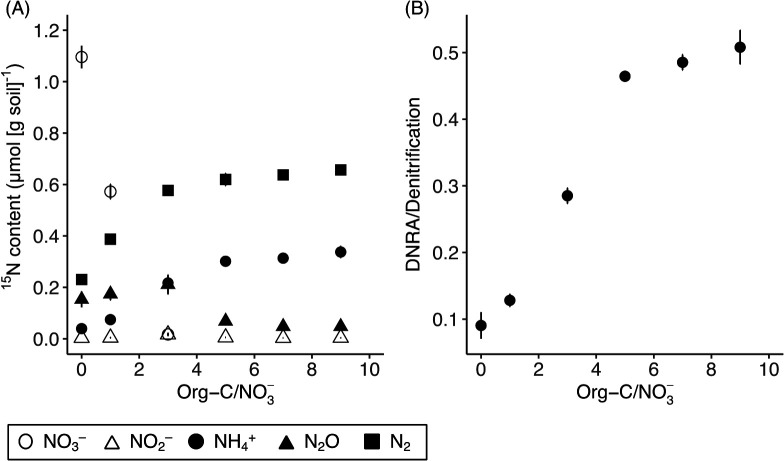
(A) Changes in ^15^N contents of NO_3_^–^, NO_2_^–^, NH_4_^+^, N_2_O, and N_2_ in the soil microcosm after a 12-hours incubation across different ratios of C-glucose and NH_4_^15^NO_3_ added (org-C/NO_3_^–^=0, 1, 3, 5, 7, and 9). (B) Relationship between org-C/NO_3_^–^ and the ratio of DNRA to denitrification. The symbol represents the average, with the bar representing the range (*n*=2).

The results from our second experiment showed that the ratio of organic carbon to NO_3_^–^ (org-C/NO_3_^–^) affected the partitioning of NO_3_^–^ reduction processes; higher org-C/NO_3_^–^ enhanced DNRA over denitrification (Fig. 2A and B). NO_3_^–^ reduction and NH_4_^+^ and N_2_ production progressed as org-C/NO_3_^–^ increased. NO_2_^–^ and N_2_O concentrations were the highest at org-C/NO_3_^–^ of 3. The ratio of DNRA to denitrification also became higher as org-C/NO_3_^–^ increased from 10% with C-glucose/NO_3_^–^ of zero, to 50% with C-glucose/NO_3_^–^ of 9. We did not detect NH_4_^+^ oxidation to NO_2_^–^ or NO_3_^–^ in soil microcosms amended with ^15^NH_4_NO_3_ and glucose or with ^15^NH_4_NO_3_ alone_._ These results indicate that DNRA is more likely to occur under NO_3_^–^ limiting (relative to org-C) environments. The ratios of DNRA to denitrification differed between the first and second experiments: the ratios of soil samples with org-C/NO_3_^–^ of 0 and 9 were larger and smaller in the second experiment, respectively. Although the reason for this result currently remains unclear, it may depend on the sampling period (see above).

### Microbial populations carrying out DNRA and denitrification

Since DNRA and denitrification both occurred simultaneously and actively in soils with org-C/NO_3_^–^ of 3, we analyzed the microbial populations responsible for these processes based on the sequences of *nrfA* transcripts for DNRA and those of *nirK* and *nirS* transcripts for denitrification. We obtained 57, 43, 45, and 46 clones for *nrfA*, *nirK* in Cluster I, *nirK* in Cluster II, and *nirS* in Cluster I, respectively. The taxonomic composition of DNRA and denitrifying microbes appeared to differ (Table 1 and S2). Many (43.9%) of the *nrfA* sequences showed the highest similarity to those from the genera *Anaeromyxobacter* and *Geobacter* (class *Deltaproteobacteria*), while many others showed the highest similarity to those from the taxa of which genus-level taxonomy is unknown. In contrast, *nirK* sequences in Cluster I showed the highest similarity to those from the genera *Bradyrhizobium* and *Afipia* in the *Bradyrhizobiaceae* family (class *Alphaproteobacteria*). *nirK* sequences in Cluster II showed the highest similarity to those from the genera *Rhodanobacter* (class *Gammaproteobacteria*) and *Ralstonia* (class *Betaproteobacteria*). *nirS* sequences showed the highest similarity with the genera *Thiothrix*, *Rhodanobacter*, and *Marinobacter* (class *Gammaproteobacteria*) and the genus *Burkholderiaceae* (class *Betaproteobacteria*). Although the majority of *nrfA*, *nirS*, and *nirK* sequences were assigned to the reference sequences with high similarity, they did not form distinct phylogenetic clades with these reference sequences with a bootstrap support of >80%, except for the‍ ‍clones assigned to the genus *Geobacter* or *Pseudogulbenkiania* (Fig. S1), which may have been due to the lack of *nrfA*, *nirS*, and *nirK* reference sequences within the database preventing accurate taxonomic assignments. Conversely, complete genome sequences within the assigned genus with *nrfA*, such as *Geobacter* and *Anaeromyxobacter*, did not possess *nirS* or *nirK*, whereas those with *nirK* or *nirS* did not possess *nrfA* (Table S3). These results suggest that the populations driving DNRA and denitrification differ, with potentially little overlap.

### Implications for DNRA and denitrification in on-site paddy soil

The results obtained herein and previous field observations in the same location suggest that DNRA is a substantial N cycling process in on-site paddy soil under practical management conditions. A previous field observation study reported that soil NO_3_^–^ and NO_2_^–^ were continuously undetectable under anoxic field conditions (Itoh *et al.*, 2013), suggesting that NO_3_^–^ and NO_2_^–^ produced by nitrification in the oxic layer are rapidly consumed by some pathway. Another field observation study that performed a meta-transcriptome analysis detected *nrfA* transcripts in greater abundance in the reduced layer than in the surface, and many of these sequences were similar to those of *Anaeromyxobacter* and *Geobacter* (Masuda *et al.*, 2017), suggesting that paddy soil in the reduced layer has the potential for DNRA largely by *Anaeromyxobacter* and *Geobacter*. By conducting controlled laboratory incubation experiments with ^15^N tracer and gene transcript-clone library analyses, we herein demonstrated that DNRA occurred in rice paddy soils at a similar rate to denitrification in NO_3_^–^-limiting (relative to organic carbon) anaerobic paddy soils and that *Anaeromyxobacter*-like and *Geobacter*-like *nrfA* expression enhanced DNRA activity. Based on these results, the paddy field may form more closed N-cycling than the nitrification-denitrification centric understanding; that is, NO_3_^–^ produced by nitrification at the surface oxidized layer is reduced not only to gaseous N_2_ via denitrification, but also to NH_4_^+^ via DNRA, below the oxidized layer.

Difficulties are associated with evaluating the extent to which DNRA and denitrification occur in on-site paddy soil because both are difficult to measure without the addition of a substrate (^15^NO_3_^–^). The contribution of DNRA to the depletion of NO_3_^–^ may be larger in an anaerobic environment with a large influx of readily degradable organic matter, but with a small influx of NO_3_^–^, leading to high org-C/NO_3_^–^. An anaerobic environment around the rhizosphere, in which a large amount of root secretions is supplied (Kuzyakov and Blagodatskaya, 2015), may be one such environment. The soil redox potential may also affect the partitioning of NO_3_^–^ reduction between DNRA and denitrification. However, a consensus has not yet been reached on the soil redox conditions under which DNRA and denitrification occur. While some studies demonstrated that DNRA occurs in more reduced soils/sediments than denitrification (Buresh and Patrick, 1978, 1981), others showed that DNRA was relatively insensitive to redox conditions (Pett-Ridge *et al.*, 2006) and even occurred in aerobic-like environments (Morley and Baggs, 2010; Yang *et al.*, 2017). Further studies are needed to identify the hotspots and hot moments‍ ‍of DNRA in on-site paddy soils (Kuzyakov and Blagodatskaya, 2015).

Rice paddy soil appears to have great potential for DNRA. Although research on DNRA in rice fields remains limited, DNRA has accounted for a significant proportion of NO_3_^–^ reduction in the paddy soils tested to date (Pandey *et al.*, 2018; 2019), with some exceptions (Shan *et al.*, 2016). The DNRA potentials of paddy soils (0.8–9.8‍ ‍μg N [g soil]^–1^ d^–1^ from our second experiment, 0.42–3.20‍ ‍μg N [g soil]^–1^ d^–1^ [Pandey *et al.*, 2018; 2019], 0.2–8.9‍ ‍μg N [g soil]^–1^ d^–1^ [Buresh and Patrick, 1978], and 0.01–0.19‍ ‍μg N [g soil]^–1^ d^–1^ [Shan *et al.*, 2016]) are often larger than those of croplands, grasslands, and forests (<1.0‍ ‍μg N [g soil]^–1^ d^–1^ [Rütting *et al.*, 2011; Putz *et al.*, 2018], except for 2.89‍ ‍μg N [g soil]^–1^ d^–1^ in a humid tropical forest [Pett-Ridge *et al.*, 2006]), although these potentials may largely depend on the experimental settings, such as the concentrations of substrates added. Moreover, *Anaeromyxobacter* and *Geobacter*, the main candidates responsible for DNRA, are widely predominant in Japanese paddy soils (Masuda *et al.*, 2017). In addition, laboratory measurements of the potential for denitrification may overestimate denitrification and underestimate DNRA because only NO_3_^–^ is added as a substrate in many cases (Tiedje *et al.*, 1989), which may enhance denitrification over DNRA. Even with the addition of organic carbon, the addition of NO_3_^–^ relieves the NO_3_^–^ limiting condition, which is also applicable to the present study. Recent studies suggested that high N fertilization suppresses DNRA, but enhances denitrification (Peng *et al.*, 2016; Pandey *et al.*, 2018; Putz *et al.*, 2018), possibly due to an increase in NO_3_^–^ availability relative to organic carbon in soils. Rice paddy fields may exhibit the more efficient use of N fertilizers than upland crop fields (Okumura, 2002; Zhou *et al.*, 2011), and strong DNRA activity in paddy soils may be the reason for this efficacy. The efficient enhancement of DNRA provides an opportunity to achieve the low-fertilization management of rice paddy fields.

## Citation

Nojiri, Y., Kaneko, Y., Azegami, Y., Shiratori, Y., Ohte, N., Senoo, K., et al. (2020) Dissimilatory Nitrate Reduction to Ammonium and Responsible Microbes in Japanese Rice Paddy Soil. *Microbes Environ ***35**: ME20069.

https://doi.org/10.1264/jsme2.ME20069

## Supplementary Material

Supplementary Material

## Figures and Tables

**Table 1.** Taxonomic candidates of the DNRA population based on *nrfA* transcripts and the denitrifying population based on *nirK* and *nirS* transcripts in the soil microcosm in a 12-hours incubation with org-C/NO_3_^–^ of 3

Gene	Phylum/Class	Genus	Proportion (%)
*nrfA* (57 clones)	*Deltaproteobacteria*	*Anaeromyxobacter*	22.8
	*Deltaproteobacteria*	*Geobacter*	21.1
	*Chloroflexi*	unidentified	15.8
	*Deltaproteobacteria*	unidentified	8.8
	*Chlorobi*	unidentified	7.0
	*Chloroflexi*	*Caldilinea*	5.3
	*Planctomycetes*	unidentified	5.3
	Others (<5%)		14.0
*nirK* Cluster I (43 clones)	*Alphaproteobacteria*	*Bradyrhizobium*	69.7
	*Alphaproteobacteria*	*Afipia*	25.5
	Others (<5%)		4.6
*nirK* Cluster II (45 clones)	*Gammaproteobacteria*	*Rhodanobacter*	15.5
	*Betaproteobacteria*	*Ralstonia*	8.8
	*Verrucomicrobia*	*Chthoniobacter*	6.6
	*Alphaproteobacteria*	*Mesorhizobium*	6.6
	Others (<5%) or unidentified		64.4
*nirS* Cluster I (46 clones)	*Gammaproteobacteria*	*Thiothrix*	28.2
	*Betaproteobacteria*	*Burkholderiaceae genus*	13.0
	*Gammaproteobacteria*	*Rhodanobacter*	10.8
	*Gammaproteobacteria*	*Marinobacter*	8.6
	*Betaproteobacteria*	*Pseudogulbenkiania*	8.6
	*Betaproteobacteria*	*Acidovorax*	6.5
	*Betaproteobacteria*	*Dechloromonas*	6.5
	*Betaproteobacteria*	*Gulbenkiania*	6.5
	Others (<5%)		10.8
