# Supplementary material for: Dissimilatory Nitrate Reduction to Ammonium and Responsible Microbes in Japanese Rice Paddy Soil
Source: Microbes Environ. 2020 Oct 6;35(4):ME20069. doi: 10.1264/jsme2.ME20069 (PMC7734399; doi:10.1264/jsme2.ME20069)
Supplement: Supplementary file 1 — Supplementary Material [file 35_20069_s1.pdf]

Table S1. PCR primers and conditions used in this study

Amplified gene	Primers	Sequence (5' -> 3')	Annealing temperature (°C)/time (second)	Reference
nrfA	nrfAF2aw	CARTGYCAYGTBGARTA	53/30	Welsh et al. (2014)
	nrfAR1	TWNGGCATRTGRCARTC		
nirK in Cluster I	nirKC1F	ATGGCGCCATCATGGTNYTNCC	56/30	Wei et al. (2015)
	nirKC1R	TCGAAGGCCTCGATNARRTTRTG		
nirK in Cluster II	nirKC2F	TGCACATCGCCAACGGNATGTWYGG	54/30	Wei et al. (2015)
	nirKC2R	GGCGCGGAAGATGSHRTGRTCNAC		
nirS in Cluster I	nirSC1F	ATCGTCAACGTCAARGARACVGG	56/30	Wei et al. (2015)
	nirSC1R	TTCGGGTGCGTCTTSABGAASAG		

Table S2. Results of a blast search of transcript sequences of *nrfA*, *nirK* in Cluster I, *nirK* in Cluster II, and *nirS* in Cluster I. We performed the homology search with BLASTP against the Fungene database using the following threshold values: E-value < 0.001 and percent identity > 60 % for the taxonomic assignment.

Gene	clone_ID	Closest_match	Identity (%)	E-value	Score	Taxon
nrfA	nrfA_1	ABC82672	76.056	7.73E-36	124	Anaeromyxobacter dehalogenans 2CP-C
	nrfA_10	ACM21454	78.947	6.14E-40	135	Geobacter daltonii FRC-32
	nrfA_11	ABQ28148	83.333	2.12E-39	134	Geobacter uraniireducens Rf4
	nrfA_12	ABC82672	91.549	1.74E-43	145	Anaeromyxobacter dehalogenans 2CP-C
	nrfA_13	BAM00537	76.119	3.98E-36	123	Caldilinea aerophila DSM 14535 = NBRC 104270
	nrfA_14	ABQ28148	78.205	4.34E-39	133	Geobacter uraniireducens Rf4
	nrfA_15	KXK52478	76.119	4.40E-35	120	Chloroflexi bacterium OLB13
	nrfA_16	ABC82672	83.099	5.73E-42	140	Anaeromyxobacter dehalogenans 2CP-C
	nrfA_17	ACM21454	79.487	7.25E-38	129	Geobacter daltonii FRC-32
	nrfA_19	ACL64314	76.119	4.65E-33	115	Anaeromyxobacter dehalogenans 2CP-1
	nrfA_20	KXK43885	70.149	2.69E-32	110	Chlorobi bacterium OLB5
	nrfA_21	KUO63373	79.104	1.64E-37	128	bacterium BRH
	nrfA_23	KPK87831	60.526	8.21E-31	110	Deltaproteobacteria bacterium SM23
	nrfA_24	KPK87831	61.842	2.14E-31	112	Deltaproteobacteria bacterium SM23
	nrfA_27	KXK43885	70.149	2.69E-32	110	Chlorobi bacterium OLB5
	nrfA_28	ACG74209	87.324	1.65E-41	139	Anaeromyxobacter sp. K
	nrfA_29	AKI98892	82.09	3.17E-37	127	Archangium gephyra
	nrfA_3	KXK52478	74.627	5.64E-34	117	Chloroflexi bacterium OLB13
	nrfA_30	KXK52478	76.119	5.59E-35	120	Chloroflexi bacterium OLB13
	nrfA_31	AJY70082	73.684	1.41E-35	123	Geobacter sulfurreducens
	nrfA_32	ACM21454	80.519	1.29E-41	140	Geobacter daltonii FRC-32
	nrfA_33	KXK43885	73.134	8.95E-36	119	Chlorobi bacterium OLB5
	nrfA_37	GAO01678	88.732	2.94E-42	141	Anaeromyxobacter sp. PSR-1
	nrfA_38	AJY70082	72.368	7.28E-35	121	Geobacter sulfurreducens
	nrfA_39	ABC82672	83.099	4.72E-39	132	Anaeromyxobacter dehalogenans 2CP-C
	nrfA_40	ABC82672	85.915	1.47E-40	137	Anaeromyxobacter dehalogenans 2CP-C
	nrfA_41	KXK52478	76.119	8.12E-35	120	Chloroflexi bacterium OLB13
	nrfA_42	KPK87831	60.526	8.21E-31	110	Deltaproteobacteria bacterium SM23
	nrfA_44	KXK52478	71.642	3.10E-32	113	Chloroflexi bacterium OLB13
	nrfA_45	QDT71785	66.667	1.33E-32	115	Planctomycetes bacterium I41
	nrfA_47	KXK52478	77.612	3.49E-36	123	Chloroflexi bacterium OLB13
	nrfA_5	No hit				
	nrfA_50	ABC82672	80.282	2.13E-37	128	Anaeromyxobacter dehalogenans 2CP-C
	nrfA_51	ABC82672	76.056	7.73E-36	124	Anaeromyxobacter dehalogenans 2CP-C
	nrfA_52	ABC82672	85.915	1.33E-41	139	Anaeromyxobacter dehalogenans 2CP-C
	nrfA_53	QDU76088	63.889	1.34E-31	112	Planctomycetes bacterium Pan97
	nrfA_54	QDT71785	66.667	1.33E-32	115	Planctomycetes bacterium I41
	nrfA_55	KPL88093	69.118	1.46E-32	114	Levilinea saccharolytica
	nrfA_56	KPK87831	61.842	2.14E-31	112	Deltaproteobacteria bacterium SM23
	nrfA_57	KXK52478	71.642	3.10E-32	113	Chloroflexi bacterium OLB13
	nrfA_60	AFH48573	79.104	1.68E-34	119	Ignavibacterium album JCM 16511
	nrfA_62	AJY70082	73.684	1.41E-35	123	Geobacter sulfurreducens
	nrfA_63	ACG74209	87.324	1.65E-41	139	Anaeromyxobacter sp. K
	nrfA_64	BAM00537	73.134	2.75E-34	118	Caldilinea aerophila DSM 14535 = NBRC 104270
	nrfA_65	KPK87831	61.842	2.14E-31	112	Deltaproteobacteria bacterium SM23
	nrfA_66	ACM21454	80.519	1.29E-41	140	Geobacter daltonii FRC-32
	nrfA_67	AXA34897	77.612	8.51E-35	120	Candidatus Sumerlaea chitinovorans
	nrfA_68	KXK43885	70.149	2.69E-32	110	Chlorobi bacterium OLB5
	nrfA_69	CUS03143	67.164	1.19E-31	111	Candidatus Promineofilum breve
	nrfA_7	BAM00537	68.657	3.89E-32	112	Caldilinea aerophila DSM 14535 = NBRC 104270
	nrfA_71	KXK52478	76.119	4.64E-35	120	Chloroflexi bacterium OLB13
	nrfA_73	ABC82672	78.873	1.16E-37	129	Anaeromyxobacter dehalogenans 2CP-C
	nrfA_74	KXK52478	74.627	5.64E-34	117	Chloroflexi bacterium OLB13
	nrfA_75	BAU23742	67.143	6.94E-29	105	Caldimicrobium thiodismutans
	nrfA_76	AJY70082	73.684	1.41E-35	123	Geobacter sulfurreducens
	nrfA_8	ABQ28148	83.333	2.12E-39	134	Geobacter uraniireducens Rf4
	nrfA_9	AJY70082	76.316	1.04E-36	126	Geobacter sulfurreducens
nirK in Cluster I	nirKC1_1	EFI52480	88.435	1.64E-97	282	Afipia sp. 1NLS2
	nirKC1_2	BAL74450	97.279	1.49E-105	302	Bradyrhizobium sp. S23321
	nirKC1_3	ABQ38713	95.238	4.48E-104	298	Bradyrhizobium sp. BTAi1
	nirKC1_4	ABC94414	93.197	1.22E-101	293	Rhizobium etli CFN 42
	nirKC1_5	CCE00380	89.116	1.99E-97	281	Bradyrhizobium sp. STM 3809

	nirKC1_6	ABQ38713	93.878	1.57E-102	294	Bradyrhizobium sp. BTAi1
	nirKC1_7	BAL74450	95.918	4.95E-104	298	Bradyrhizobium sp. S23321
	nirKC1_8	CCE00380	89.116	7.46E-97	280	Bradyrhizobium sp. STM 3809
	nirKC1_9	CCE00380	87.075	1.96E-94	274	Bradyrhizobium sp. STM 3809
	nirKC1_10	CCE00380	86.395	4.67E-93	270	Bradyrhizobium sp. STM 3809
	nirKC1_11	CCE00380	88.435	4.18E-97	281	Bradyrhizobium sp. STM 3809
	nirKC1_12	CCE00380	88.435	4.18E-97	281	Bradyrhizobium sp. STM 3809
	nirKC1_13	CCE00380	89.796	4.99E-98	283	Bradyrhizobium sp. STM 3809
	nirKC1_14	BAL74450	96.599	1.32E-104	300	Bradyrhizobium sp. S23321
	nirKC1_15	CCE00380	89.116	1.71E-96	279	Bradyrhizobium sp. STM 3809
	nirKC1_16	EFI52480	88.435	1.64E-97	282	Afipia sp. 1NLS2
	nirKC1_17	EFI52480	88.435	6.22E-98	283	Afipia sp. 1NLS2
	nirKC1_18	BAL74450	97.279	1.49E-105	302	Bradyrhizobium sp. S23321
	nirKC1_19	ABQ38713	93.197	1.96E-101	291	Bradyrhizobium sp. BTAi1
	nirKC1_20	CCE00380	88.435	2.31E-95	276	Bradyrhizobium sp. STM 3809
	nirKC1_21	EIM72710	80.822	2.19E-88	258	Nitratireductor aquibiodomus RA22
	nirKC1_22	EFI52480	88.435	1.64E-97	282	Afipia sp. 1NLS2
	nirKC1_23	EFI52480	88.435	1.64E-97	282	Afipia sp. 1NLS2
	nirKC1_24	BAL74450	95.918	1.13E-103	297	Bradyrhizobium sp. S23321
	nirKC1_25	CCD88817	98.639	2.15E-106	304	Bradyrhizobium sp. ORS 285
	nirKC1_26	CCE00380	89.116	1.53E-97	281	Bradyrhizobium sp. STM 3809
	nirKC1_27	EFI52480	85.714	1.08E-94	275	Afipia sp. 1NLS2
	nirKC1_28	CCD88817	98.639	2.15E-106	304	Bradyrhizobium sp. ORS 285
	nirKC1_29	ABQ38713	87.755	8.79E-93	270	Bradyrhizobium sp. BTAi1
	nirKC1_30	CCE00380	88.435	6.97E-96	277	Bradyrhizobium sp. STM 3809
	nirKC1_31	ABQ38713	87.755	8.79E-93	270	Bradyrhizobium sp. BTAi1
	nirKC1_32	EFI52480	88.435	1.64E-97	282	Afipia sp. 1NLS2
	nirKC1_33	ABQ38713	87.755	7.97E-93	270	Bradyrhizobium sp. BTAi1
	nirKC1_34	CCE00380	89.796	4.99E-98	283	Bradyrhizobium sp. STM 3809
	nirKC1_35	CCE00380	89.796	4.99E-98	283	Bradyrhizobium sp. STM 3809
	nirKC1_36	BAL74450	96.599	5.82E-105	300	Bradyrhizobium sp. S23321
	nirKC1_37	EFI52480	85.714	4.82E-96	278	Afipia sp. 1NLS2
	nirKC1_38	EFI52480	87.755	8.42E-97	280	Afipia sp. 1NLS2
	nirKC1_39	EFI52480	88.435	1.64E-97	282	Afipia sp. 1NLS2
	nirKC1_40	CCE00380	88.435	4.18E-97	281	Bradyrhizobium sp. STM 3809
	nirKC1_41	EFI52480	87.755	7.64E-97	280	Afipia sp. 1NLS2
	nirKC1_42	CCE00380	89.116	1.53E-97	281	Bradyrhizobium sp. STM 3809
	nirKC1_43	CCE00380	89.796	5.81E-98	283	Bradyrhizobium sp. STM 3809
nirK in Cluster II	nirKC2_1	No hit				
	nirKC2_2	EDY19063	82.482	2.54E-78	237	Chthoniobacter flavus Ellin428
	nirKC2_3	No hit				
	nirKC2_4	No hit				
	nirKC2_5	No hit				
	nirKC2_6	CBJ52550	76.471	3.36E-74	226	Ralstonia solanacearum PSI07
	nirKC2_7	ACU06855	63.704	6.08E-57	177	Flavobacteriaceae bacterium 3519-10
	nirKC2_8	No hit				
	nirKC2_9	EGM25430	67.391	1.26E-64	199	Salinisphaera shabanensis E1L3A
	nirKC2_10	No hit				
	nirKC2_11	AER51969	74.265	8.05E-71	217	Ralstonia pickettii
	nirKC2_12	EHK55601	68.345	2.01E-65	197	Mesorhizobium alhagi CCNWXJ12-2
	nirKC2_13	AVL50482	77.206	9.70E-74	224	Moraxella catarrhalis
	nirKC2_14	EIL98512	79.412	1.02E-75	228	Rhodanobacter thiooxydans LCS2
	nirKC2_15	EHK55601	69.065	5.81E-66	198	Mesorhizobium alhagi CCNWXJ12-2
	nirKC2_16	No hit				
	nirKC2_17	No hit				
	nirKC2_18	ADE28552	64.179	3.46E-57	182	Rhodanobacter denitrificans
	nirKC2_19	CBJ52550	76.471	5.39E-72	220	Ralstonia solanacearum PSI07
	nirKC2_20	ABM36641	71.223	6.51E-64	198	Polaromonas naphthalenivorans CJ2
	nirKC2_21	EDY19063	78.102	8.91E-74	225	Chthoniobacter flavus Ellin428
	nirKC2_22	No hit				
	nirKC2_23	No hit				
	nirKC2_24	No hit				
	nirKC2_25	CBJ52550	76.471	3.36E-74	226	Ralstonia solanacearum PSI07
	nirKC2_26	EIL91558	76.471	9.73E-74	224	Rhodanobacter fulvus Jip2
	nirKC2_27	ACZ37592	62.774	2.08E-52	166	Sphaerobacter thermophilus DSM 20745
	nirKC2_28	No hit				

	nirKC2_29	EIL91558	76.471	9.73E-74	224	Rhodanobacter fulvus Jip2
	nirKC2_30	EHK55601	69.065	7.30E-66	198	Mesorhizobium alhagi CCNWXJ12-2
	nirKC2_31	ABD46561	68.462	1.98E-58	179	Vermamoeba vermiformis
	nirKC2_32	EIL91558	76.471	9.73E-74	224	Rhodanobacter fulvus Jip2
	nirKC2_33	No hit				
	nirKC2_34	No hit				
	nirKC2_35	EIL91558	76.471	9.73E-74	224	Rhodanobacter fulvus Jip2
	nirKC2_36	ACV27434	75.735	4.50E-72	219	Kangiella koreensis DSM 16069
	nirKC2_37	BAI75496	97.08	2.81E-94	273	Azospirillum sp. B510
	nirKC2_38	No hit				
	nirKC2_39	EDY19063	78.102	1.34E-73	224	Chthoniobacter flavus Ellin428
	nirKC2_40	ABX03943	67.407	4.61E-61	191	Herpetosiphon aurantiacus DSM 785
	nirKC2_41	EIL91558	76.471	9.73E-74	224	Rhodanobacter fulvus Jip2
	nirKC2_42	No hit				
	nirKC2_43	No hit				
	nirKC2_44	EGM25430	63.768	9.91E-60	187	Salinisphaera shabanensis E1L3A
	nirKC2_45	ACM53116	69.118	5.04E-66	200	Chloroflexus sp. Y-400-fl
nirS in Cluster I	nirSC1_1	ABG36514	75.207	2.07E-65	194	Marinobacter sp. CG157051
	nirSC1_2	CUA82351	82.645	7.84E-69	212	Gulbenkiania indica
	nirSC1_3	BAH90680	83.607	1.85E-74	217	Rhodanobacter sp. D206a
	nirSC1_4	BAH90677	85.95	4.48E-74	216	Burkholderiaceae bacterium N52
	nirSC1_5	AGO45492	72.727	3.03E-65	194	Thiothrix lacustris
	nirSC1_6	AGO45492	72.727	3.03E-65	194	Thiothrix lacustris
	nirSC1_7	AGO45492	74.38	4.79E-65	194	Thiothrix lacustris
	nirSC1_8	ABG36514	75.207	2.07E-65	194	Marinobacter sp. CG157051
	nirSC1_9	EEG09987	100	1.60E-84	252	Pseudogulbenkiania ferrooxidans 2002
	nirSC1_10	BAH90677	76.033	3.78E-69	204	Burkholderiaceae bacterium N52
	nirSC1_11	AGO45492	71.901	2.22E-63	189	Thiothrix lacustris
	nirSC1_12	AGO45492	71.901	2.22E-63	189	Thiothrix lacustris
	nirSC1_13	CAK32529	91.2	8.21E-83	238	Simplicispira psychrophila
	nirSC1_14	BAH90677	85.124	6.35E-74	216	Burkholderiaceae bacterium N52
	nirSC1_15	ABG36514	75.207	2.07E-65	194	Marinobacter sp. CG157051
	nirSC1_16	EEG09987	100	1.60E-84	252	Pseudogulbenkiania ferrooxidans 2002
	nirSC1_17	BAH90680	91.803	3.73E-81	234	Rhodanobacter sp. D206a
	nirSC1_18	CUA82351	82.645	7.84E-69	212	Gulbenkiania indica
	nirSC1_19	BAL25833	85.124	5.36E-72	220	Azoarcus sp. KH32C
	nirSC1_20	ABG36514	75.207	2.07E-65	194	Marinobacter sp. CG157051
	nirSC1_21	KXB30410	96	7.74E-84	250	Dechloromonas denitrificans
	nirSC1_22	EER61763	78.947	9.39E-63	196	Acidovorax delafieldii 2AN
	nirSC1_23	BAH90677	84.298	5.95E-74	216	Burkholderiaceae bacterium N52
	nirSC1_24	BAH90680	91.803	3.73E-81	234	Rhodanobacter sp. D206a
	nirSC1_25	EEG09987	100	1.60E-84	252	Pseudogulbenkiania ferrooxidans 2002
	nirSC1_26	AGO45492	72.727	3.03E-65	194	Thiothrix lacustris
	nirSC1_27	AGO45492	71.901	6.86E-65	193	Thiothrix lacustris
	nirSC1_28	KPJ79435	84.298	1.67E-71	218	Gammaproteobacteria bacterium SG8
	nirSC1_29	AAZ48052	99.174	1.06E-83	250	Dechloromonas aromatica RCB
	nirSC1_30	BAH90677	97.521	3.57E-85	244	Burkholderiaceae bacterium N52
	nirSC1_31	EER61763	80.992	4.29E-69	213	Acidovorax delafieldii 2AN
	nirSC1_32	AGO45492	71.93	2.92E-60	181	Thiothrix lacustris
	nirSC1_33	AGO45492	73.554	3.71E-67	199	Thiothrix lacustris
	nirSC1_34	AGO45492	71.901	2.22E-63	189	Thiothrix lacustris
	nirSC1_35	AAZ48052	99.174	1.54E-83	250	Dechloromonas aromatica RCB
	nirSC1_36	BAL25833	80.992	9.13E-69	211	Azoarcus sp. KH32C
	nirSC1_37	CUA82351	82.645	7.84E-69	212	Gulbenkiania indica
	nirSC1_38	AGO45492	71.901	2.22E-63	189	Thiothrix lacustris
	nirSC1_39	BAH90680	81.148	8.69E-76	221	Rhodanobacter sp. D206a
	nirSC1_40	EEG09987	100	1.60E-84	252	Pseudogulbenkiania ferrooxidans 2002
	nirSC1_41	AGO45492	70.248	1.31E-61	185	Thiothrix lacustris
	nirSC1_42	CBG92407	86.777	3.00E-77	225	Dechlorospirillum sp. I-Bh37-22
	nirSC1_43	EER61763	80.992	4.29E-69	213	Acidovorax delafieldii 2AN
	nirSC1_44	AGO45492	72.727	3.03E-65	194	Thiothrix lacustris
	nirSC1_45	BAH90677	85.124	3.38E-73	214	Burkholderiaceae bacterium N52
	nirSC1_46	BAH90680	90.164	1.03E-80	233	Rhodanobacter sp. D206a

Table S3. Presence of *nrfA*, *nirK*, and *nirS* within the deposited complete genome sequences in KEGG database belonging to the assigned genus in **Table 1**. The genomes that possess either of the genes are displayed. ID after the gene name refers to the gene ID of KEGG.

Genus	Species	*nrfA* [K03385]	*nirK* [K00368]	*nirS* [K15864]
Anaeromyxobacter	dehalogenans 2CP-C	X		
	sp. Fw109-5	X		
	sp. K	X		
Geobacter	sulfurreducens PCA	X		
	sulfurreducens KN400	X		
	metallireducens	X		
	uraniireducens	X		
	lovleyi	X		
	bemidjiensis	X		
	daltonii FRC-32	X		
	sp. M21	X		
	sp. M18	X		
	pickeringii	X		
	anodireducens	X		
Caldilinea	aerophila	X		
Bradyrhizobium	diazoefficiens USDA 110		X	
	japonicum USDA 6		X	
	japonicum E109		X	
	sp. ORS 278		X	
	sp. BTAi1		X	
	sp. S23321		X	
	oligotrophicum		X	
	sp. CCGE-LA001		X	
	sp. BF49		X	
	icense		X	
	sp. ORS 285		X	
	ottawaense		X	
	amphicarpaeae		X	
	guangdongense		X	
	guangzhouense		X	
	symbiodeficiens		X	
	betae		X	
Rhodanobacter	denitrificans		X	
	glycinis		X	
Ralstonia	solanacearum GMI1000		X	
	solanacearum PSI07		X	
	solanacearum Po82		X	
	solanacearum CMR15		X	
	solanacearum FQY_4		X	
	solanacearum UY031		X	
	pickettii 12J		X	
	pickettii 12D		X	
	pseudosolanacearum		X	
Mesorhizobium	loti NZP2037		X	
	ciceri (biovar Biserrulae)		X	
	opportunistum		X	
	australicum		X	
	amorphae		X	
	sp. M9A.F.Ca.ET.002.03.1.2		X	
Marinobacter	sp. BSs20148			X
	psychrophilus			X
	Pseudogulbenkiania sp. NH8B			X
Acidovorax	sp. JS42			X
	ebreus			X
	carolinensis NA2			X
	carolinensis P4			X
	carolinensis NA3			X
	sp. 1608163			X
Dechloromonas	aromatica			X
	sp. HYN0024			X

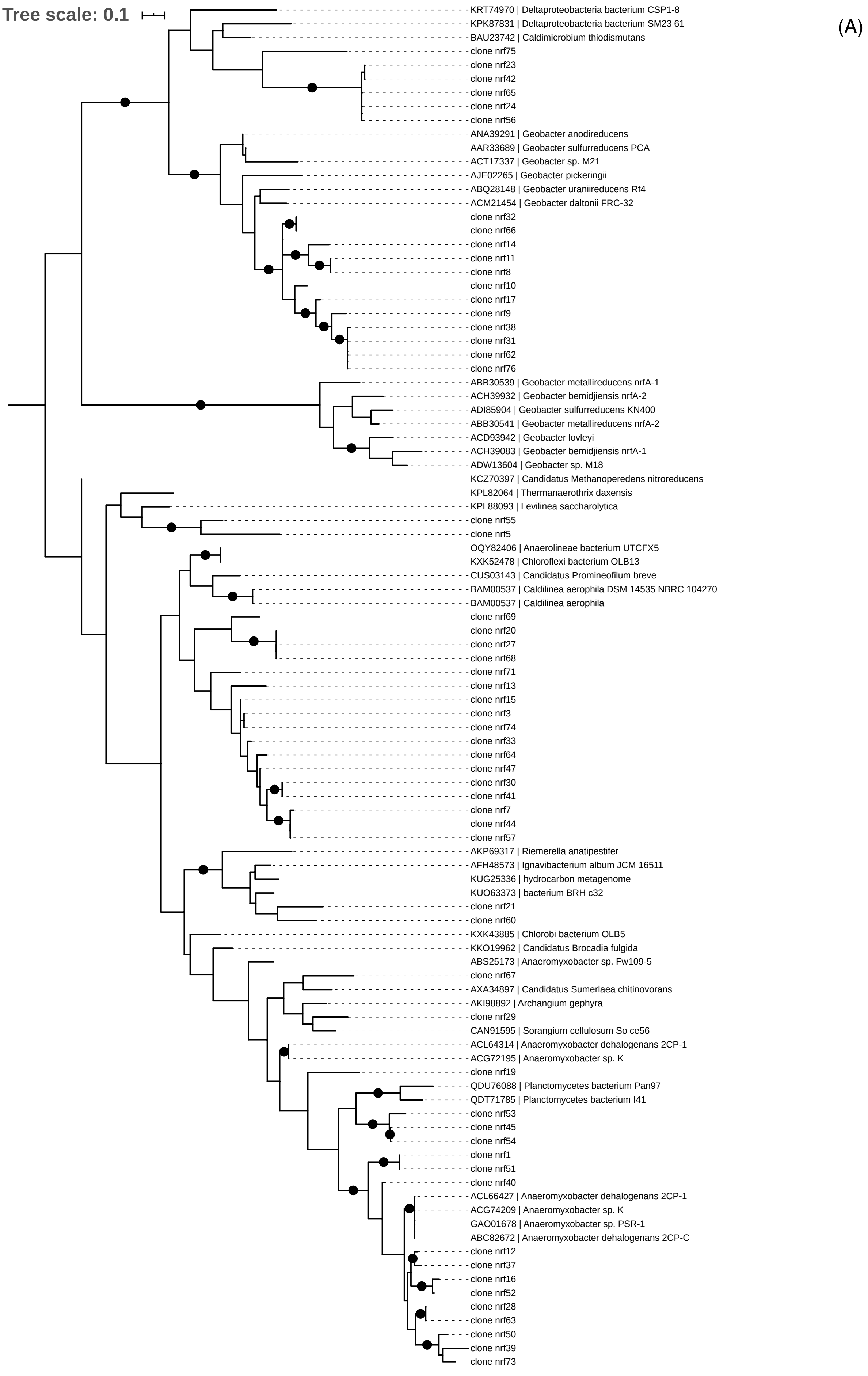

Tree scale: 0.1
(A)
KRT74970 | Deltaproteobacteria bacterium CSP1-8
KPK87831 | Deltaproteobacteria bacterium SM23 61
BAU23742 | Caldimicrobium thiodismutans
clone nrf75
clone nrf23
clone nrf42
clone nrf65
clone nrf24
clone nrf56
ANA39291 | Geobacter anodireducens
AAR33689 | Geobacter sulfurreducens PCA
ACT17337 | Geobacter sp. M21
AJE02265 | Geobacter pickeringii
ABQ28148 | Geobacter uraniireducens Rf4
ACM21454 | Geobacter daltonii FRC-32
clone nrf32
clone nrf66
clone nrf14
clone nrf11
clone nrf8
clone nrf10
clone nrf17
clone nrf9
clone nrf38
clone nrf31
clone nrf62
clone nrf76
ABB30539 | Geobacter metallireducens nrfA-1
ACH39932 | Geobacter bemidjiensis nrfA-2
ADI85904 | Geobacter sulfurreducens KN400
ABB30541 | Geobacter metallireducens nrfA-2
ACD93942 | Geobacter lovleyi
ACH39083 | Geobacter bemidjiensis nrfA-1
ADW13604 | Geobacter sp. M18
KCZ70397 | Candidatus Methanoperedens nitroreducens
KPL82064 | Thermanaerothrix daxensis
KPL88093 | Levilinea saccharolytica
clone nrf55
clone nrf5
OQY82406 | Anaerolineae bacterium UTCFX5
KXK52478 | Chloroflexi bacterium OLB13
CUS03143 | Candidatus Promineofilum breve
BAM00537 | Caldilinea aerophila DSM 14535 NBRC 104270
BAM00537 | Caldilinea aerophila
clone nrf69
clone nrf20
clone nrf27
clone nrf68
clone nrf71
clone nrf13
clone nrf15
clone nrf3
clone nrf74
clone nrf33
clone nrf64
clone nrf47
clone nrf30
clone nrf41
clone nrf7
clone nrf44
clone nrf57
AKP69317 | Riemerella anatipestifer
AFH48573 | Ignavibacterium album JCM 16511
KUG25336 | hydrocarbon metagenome
KUO63373 | bacterium BRH c32
clone nrf21
clone nrf60
KXK43885 | Chlorobi bacterium OLB5
KKO19962 | Candidatus Brocadia fulgida
ABS25173 | Anaeromyxobacter sp. Fw109-5
clone nrf67
AXA34897 | Candidatus Sumerlaea chitinovorans
AKI98892 | Archangium gephyra
clone nrf29
CAN91595 | Sorangium cellulosum So ce56
ACL64314 | Anaeromyxobacter dehalogenans 2CP-1
ACG72195 | Anaeromyxobacter sp. K
clone nrf19
QDU76088 | Planctomycetes bacterium Pan97
QDT71785 | Planctomycetes bacterium I41
clone nrf53
clone nrf45
clone nrf54
clone nrf1
clone nrf51
clone nrf40
ACL66427 | Anaeromyxobacter dehalogenans 2CP-1
ACG74209 | Anaeromyxobacter sp. K
GAO01678 | Anaeromyxobacter sp. PSR-1
ABC82672 | Anaeromyxobacter dehalogenans 2CP-C
clone nrf12
clone nrf37
clone nrf16
clone nrf52
clone nrf28
clone nrf63
clone nrf50
clone nrf39
clone nrf73

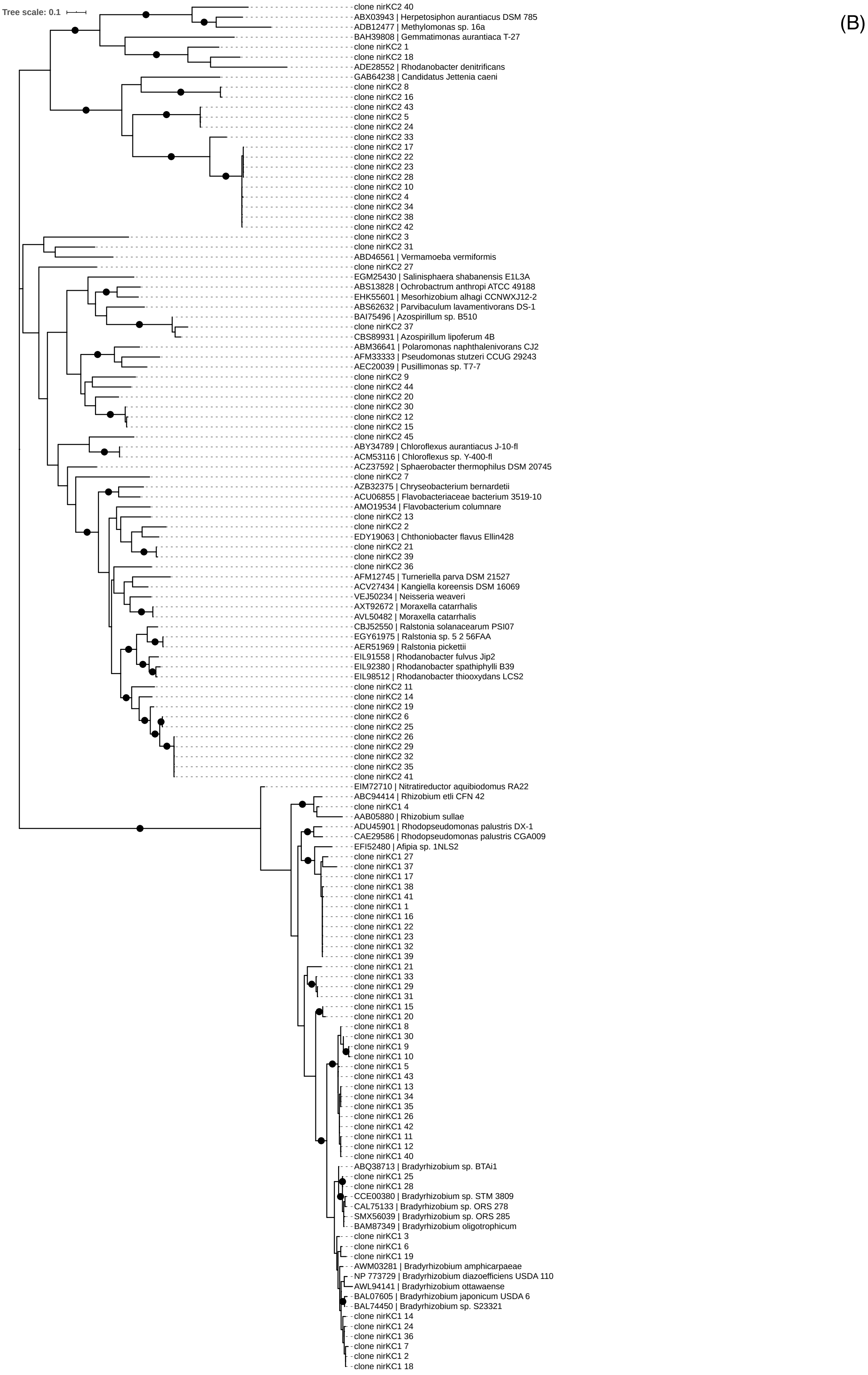
Tree scale: 0.1
(B)
clone nirKC2 40
ABX03943 | Herpetosiphon aurantiacus DSM 785
ADB12477 | Methylomonas sp. 16a
BAH39808 | Gemmatimonas aurantiaca T-27
clone nirKC2 1
clone nirKC2 18
ADE28552 | Rhodanobacter denitrificans
GAB64238 | Candidatus Jettenia caeni
clone nirKC2 8
clone nirKC2 16
clone nirKC2 43
clone nirKC2 5
clone nirKC2 24
clone nirKC2 33
clone nirKC2 17
clone nirKC2 22
clone nirKC2 23
clone nirKC2 28
clone nirKC2 10
clone nirKC2 4
clone nirKC2 34
clone nirKC2 38
clone nirKC2 42
clone nirKC2 3
clone nirKC2 31
ABD46561 | Vermamoeba vermiformis
clone nirKC2 27
EGM25430 | Salinisphaera shabanensis E1L3A
ABS13828 | Ochrobactrum anthropi ATCC 49188
EHK55601 | Mesorhizobium alhagi CCNWXJ12-2
ABS62632 | Parvibaculum lavamentivorans DS-1
BAI75496 | Azospirillum sp. B510
clone nirKC2 37
CBS89931 | Azospirillum lipoferum 4B
ABM36641 | Polaromonas naphthalenivorans CJ2
AFM33333 | Pseudomonas stutzeri CCUG 29243
AEC20039 | Pusillimonas sp. T7-7
clone nirKC2 9
clone nirKC2 44
clone nirKC2 20
clone nirKC2 30
clone nirKC2 12
clone nirKC2 15
clone nirKC2 45
ABY34789 | Chloroflexus aurantiacus J-10-fl
ACM53116 | Chloroflexus sp. Y-400-fl
ACZ37592 | Sphaerobacter thermophilus DSM 20745
clone nirKC2 7
AZB32375 | Chryseobacterium bernardetii
ACU06855 | Flavobacteriaceae bacterium 3519-10
AMO19534 | Flavobacterium columnare
clone nirKC2 13
clone nirKC2 2
EDY19063 | Chthoniobacter flavus Ellin428
clone nirKC2 21
clone nirKC2 39
clone nirKC2 36
AFM12745 | Turneriella parva DSM 21527
ACV27434 | Kangiella koreensis DSM 16069
VEJ50234 | Neisseria weaveri
AXT92672 | Moraxella catarrhalis
AVL50482 | Moraxella catarrhalis
CBJ52550 | Ralstonia solanacearum PSI07
EGY61975 | Ralstonia sp. 5 2 56FAA
AER51969 | Ralstonia pickettii
EIL91558 | Rhodanobacter fulvus Jip2
EIL92380 | Rhodanobacter spathiphylli B39
EIL98512 | Rhodanobacter thiooxydans LCS2
clone nirKC2 11
clone nirKC2 14
clone nirKC2 19
clone nirKC2 6
clone nirKC2 25
clone nirKC2 26
clone nirKC2 29
clone nirKC2 32
clone nirKC2 35
clone nirKC2 41
EIM72710 | Nitratireductor aquibiodomus RA22
ABC94414 | Rhizobium etli CFN 42
clone nirKC1 4
AAB05880 | Rhizobium sullae
ADU45901 | Rhodopseudomonas palustris DX-1
CAE29586 | Rhodopseudomonas palustris CGA009
EFI52480 | Afipia sp. 1NLS2
clone nirKC1 27
clone nirKC1 37
clone nirKC1 17
clone nirKC1 38
clone nirKC1 41
clone nirKC1 1
clone nirKC1 16
clone nirKC1 22
clone nirKC1 23
clone nirKC1 32
clone nirKC1 39
clone nirKC1 21
clone nirKC1 33
clone nirKC1 29
clone nirKC1 31
clone nirKC1 15
clone nirKC1 20
clone nirKC1 8
clone nirKC1 30
clone nirKC1 9
clone nirKC1 10
clone nirKC1 5
clone nirKC1 43
clone nirKC1 13
clone nirKC1 34
clone nirKC1 35
clone nirKC1 26
clone nirKC1 42
clone nirKC1 11
clone nirKC1 12
clone nirKC1 40
ABQ38713 | Bradyrhizobium sp. BTAi1
clone nirKC1 25
clone nirKC1 28
CCE00380 | Bradyrhizobium sp. STM 3809
CAL75133 | Bradyrhizobium sp. ORS 278
SMX56039 | Bradyrhizobium sp. ORS 285
BAM87349 | Bradyrhizobium oligotrophicum
clone nirKC1 3
clone nirKC1 6
clone nirKC1 19
AWM03281 | Bradyrhizobium amphicarpaeae
NP 773729 | Bradyrhizobium diazoefficiens USDA 110
AWL94141 | Bradyrhizobium ottawaense
BAL07605 | Bradyrhizobium japonicum USDA 6
BAL74450 | Bradyrhizobium sp. S23321
clone nirKC1 14
clone nirKC1 24
clone nirKC1 36
clone nirKC1 7
clone nirKC1 2
clone nirKC1 18

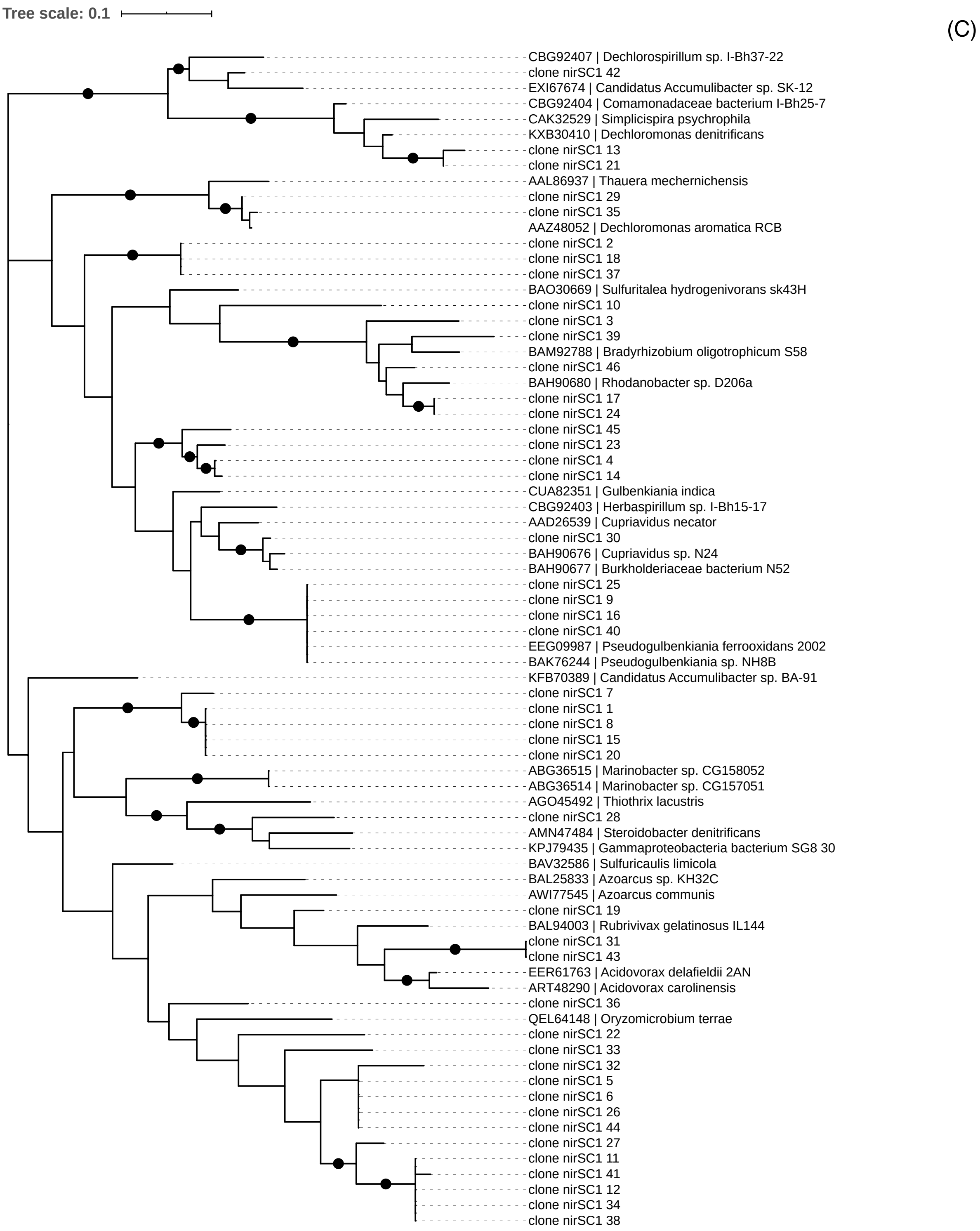

Supplementary figure 1. Maximum likelihood phylogenetic trees based on the *nrfA* (A), *nirK* (B) and *nirS* (C) amino acid sequences. Bootstrap values greater than 80% are indicated with dots at nodes. Names beginning with clone denote sequences obtained in this study. The reference sequences are included, with the accession numbers in DDBJ/EMBL/GenBank and taxon names.
